# Nutritional Value, Phytochemical Potential, and Therapeutic Benefits of Pumpkin (*Cucurbita* sp.)

**DOI:** 10.3390/plants11111394

**Published:** 2022-05-24

**Authors:** Maria Batool, Muhammad Modassar Ali Nawaz Ranjha, Ume Roobab, Muhammad Faisal Manzoor, Umar Farooq, Hafiz Rehan Nadeem, Muhammad Nadeem, Rabia Kanwal, Hamada AbdElgawad, Soad K. Al Jaouni, Samy Selim, Salam A. Ibrahim

**Affiliations:** 1 University Institute of Diet and Nutritional Sciences, University of Lahore, Gujrat 50700, Pakistan; mbatool215@gmail.com; 2 Institute of Food Science and Nutrition, University of Sargodha, Sargodha 40100, Pakistan; modassarranjha@gmail.com (M.M.A.N.R.); mnadeemft@gmail.com (M.N.); 3 School of Food Science and Engineering, South China University of Technology, Guangzhou 510641, China; mahroba73@gmail.com (U.R.); kanwalrabia043@gmail.com (R.K.); 4 School of Food and Biological Engineering, Jiangsu University, Zhenjiang 212013, China; faisaluos26@gmail.com; 5 Department of Food Science and Technology, Muhammad Nawaz Shareef University of Agriculture, Multan 59300, Pakistan; umar.farooq@mnsuam.edu.pk; 6 Institute of Food Science and Nutrition, Bahauddin Zakariya University, Multan 59300, Pakistan; hrnfoodscientist@gmail.com; 7 Integrated Molecular Plant Physiology Research, Department of Biology, University of Antwerp, 2020 Antwerpen, Belgium; hamada.abdelgawad@uantwerpen.be; 8 Botany and Microbiology Department, Faculty of Science, Beni-Suef University, Beni-Suef 62511, Egypt; 9 Department of Hematology/Oncology, Yousef Abdulatif Jameel Scientific Chair of Prophetic Medicine Application, Faculty of Medicine, King Abdulaziz University, Jeddah 21589, Saudi Arabia; saljaouni@kau.edu.sa; 10 Department of Clinical Laboratory Sciences, College of Applied Medical Sciences, Jouf University, Sakaka 72388, Saudi Arabia; 11 Food Microbiology and Biotechnology Laboratory, North Carolina Agricultural and Technical State University, Greensboro, NC 27411, USA

**Keywords:** pumpkin, phytochemicals, bioactive compounds, therapeutic potential

## Abstract

Pumpkin is a well-known multifunctional ingredient in the diet, full of nutrients, and has opened new vistas for scientists during the past years. The fruit of pumpkin including the flesh, seed, and peel are a rich source of primary and secondary metabolites, including proteins, carbohydrates, monounsaturated fatty acids, polyunsaturated fatty acids, carotenoids, tocopherols, tryptophan, delta-7-sterols, and many other phytochemicals. This climber is traditionally used in many countries, such as Austria, Hungary, Mexico, Slovenia, China, Spain, and several Asian and African countries as a functional food and provides health promising properties. Other benefits of pumpkin, such as improving spermatogenesis, wound healing, antimicrobial, anti-inflammatory, antioxidative, anti-ulcerative properties, and treatment of benign prostatic hyperplasia have also been confirmed by researchers. For better drug delivery, nanoemulsions and niosomes made from pumpkin seeds have also been reported as a health promising tool, but further research is still required in this field. This review mainly focuses on compiling and summarizing the most relevant literature to highlight the nutritional value, phytochemical potential, and therapeutic benefits of pumpkin.

## 1. Introduction

Products from natural sources have been used for centuries as functional and nutraceutical foods [1,2,3,4,5,6]. During the past years, scientists have been working on understanding the molecular-level effects of various nutrients on several chronic and deadly diseases [7,8,9]. People can adjust to different climate and habitat changes due to a wide range of nutrients that alter multiple genes’ natural mode at the molecular level [10]. In other words, molecular changes are due to nutrients. Keeping this in mind, researchers are engaged in preventing and treating diseases with the use of pertinent food(s) rather than medicines. Moreover, studies have demonstrated that healthy eating is a reasonable and economical method to treat diseases [11].

Pumpkin has attracted increasing attention from scientists due to its nutritional profile. It is a nutritious and economical product and belongs to the Cucurbitaceae family. *Cucurbita pepo* L., *Cucurbita maxima Duchesne*, and *Cucurbita moschata Duchesne* are harvested worldwide due to their economical and environmentally friendly properties [12]. In many countries, pumpkin is used as a medicine for its anti-inflammatory, antioxidant, antiviral, and antidiabetic properties, particularly in Austria, Hungary, Mexico, Slovenia, China, Spain, and various European, Asian, and African countries [13]. Worldwide, pumpkin is harvested for its peel, flesh, and seeds. The seeds are usually large in size with a high content of polyunsaturated and monounsaturated fatty acids. Linoleic acid, oleic acid, palmitic acid, ECN-44, ECN-46, tocopherols, ß-sitosterol, and delta-7-sterols constitute a large quantity of pumpkin seed oil [14].

For decades, several research studies have been conducted on the active ingredients of pumpkin peel, flesh, and seeds to provide a thumbnail sketch of their health-related impacts, which have demonstrated its anti-inflammatory [15], antibacterial [16], anticarcinogenic [17], antidiabetic [18], and antihypertensive properties, associated with this climber for diabetes [19].

**Figure 1:**
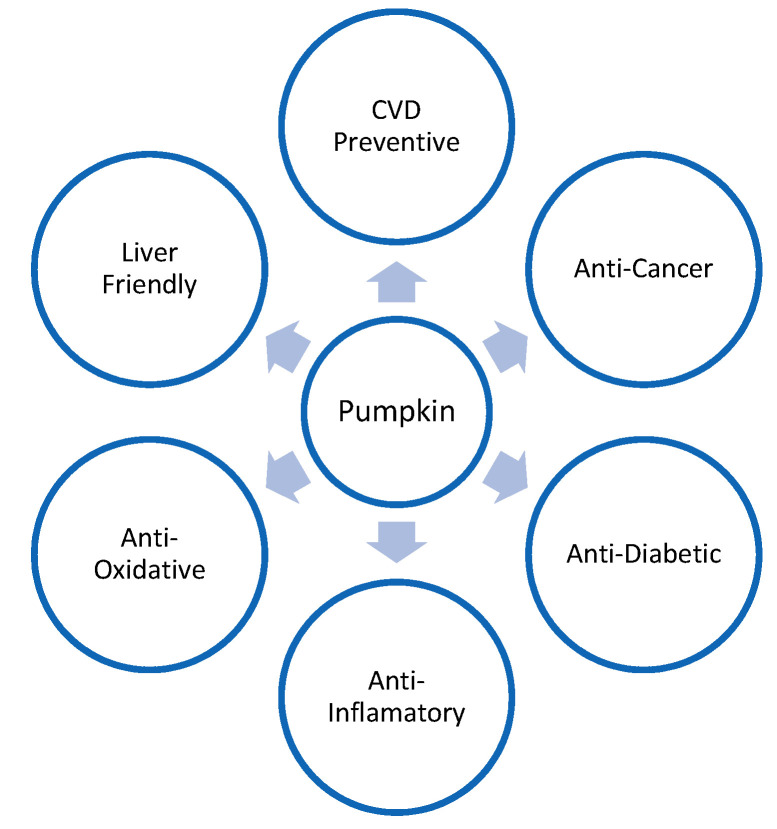
Health-friendly properties of pumpkin.

Figure 1 illustrates the health-friendly properties of pumpkins.

This review article focuses on compiling all of the research related to the nutritional composition of pumpkin peel, flesh, and seeds as well as their health-related benefits, involving scavenging activities against many chronic diseases. Safety concerns in regard to the use of pumpkins in some situations and in vulnerable groups are also discussed. In addition, recommendations for future research are proposed at the end of this paper.

## 2. Composition of Cucurbita

All foodstuffs are regarded as food supplements, although the term nutraceutical is typically used to describe goods that have not been properly evaluated for their health-related benefits on the market. Considering that pumpkin is a good source of nutrients, it may be regarded as nutraceutical [20]. The authors of [21] reported that the shape, size, flavor, color, and nutritional contents of pumpkin have genetic variation in different parts of the world. In addition, their nutrient composition will differ depending on their origins and cultivation environments (i.e., *C. maxima*, *C. pepo*, *C. moschata*). Moreover, research has demonstrated that pumpkin seeds and flesh are significant sources of proteins, carotenoids, tocopherols, and antioxidants and are low-caloric [22].

**Figure 2:**
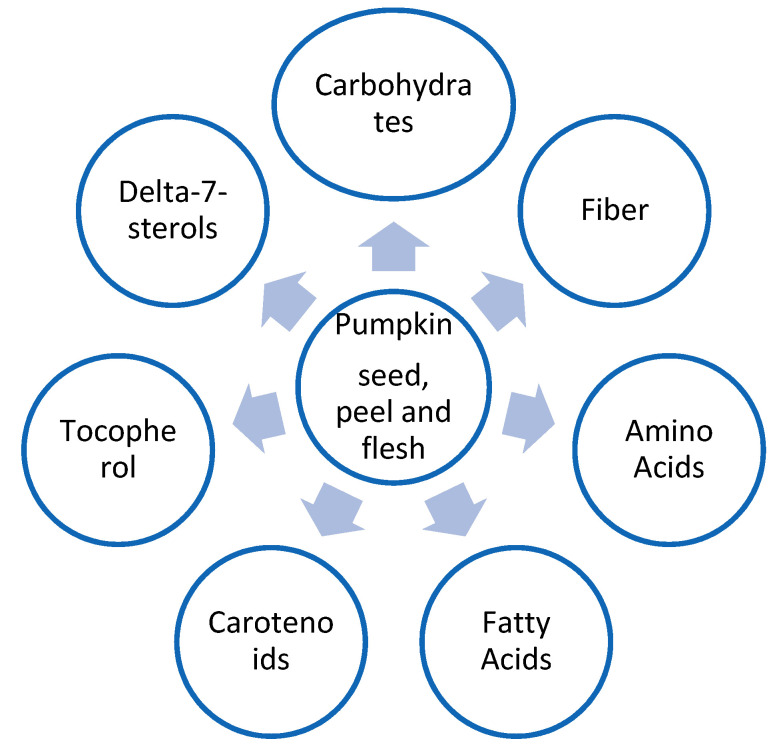
Nutritional composition of pumpkin seeds, peel, and flesh.

Figure 2 illustrates the nutritional composition of pumpkin seeds, peel, and flesh.

In 2006, a study found that pumpkin peel contains alcohol insoluble polysaccharides (AIP), which tend to attenuate bile acid and support the growth of gut microbiota [23]. Moreover, in a study on snacks made from pumpkins, it was stated that industries focus on the flesh and discard the seeds and peel as wastage (18–21%) [24].

Pumpkin fruit and its derivative products have become more popular in agriculture, medicines, and food processing due to their nutritious and health-promoting properties. Of note, the chemical composition of pumpkin varies across species and cultivars grown in various regions [25]. Pulp and seeds are the main components of pumpkin. Polysaccharides, colors, amino acids, active proteins, and minerals are found in pumpkin pulp. They are an excellent supply of potassium, phosphate, and magnesium as well as a good supply of lipids and proteins [26,27].

Proteins, carotene, mineral salts, vitamins, polysaccharides, such as pectin, as well as other elements, such as phenolic compounds and terpenoids are found in pumpkin pulp, which have both nutritional and health-protective values.

Minerals required by the human body are obtained through the regular consumption of food. Minerals play vital roles in a variety of bodily processes. Several minerals essential to human health may be found in pumpkins. Phytosterols and phytonutrients are found in abundance in pumpkin pulp [28,29]. Carotenoids, tocopherols, and sterols found in pumpkin-derived products have a wide range of biological action, as demonstrated by in vivo studies [20].

Bioactive chemicals found in pumpkin seeds, which are typically regarded as agricultural waste, have fascinating nutraceutical qualities [20]. The elements zinc, phosphorous, magnesium, potassium, and selenium found in pumpkin seeds make them a nutritional powerhouse and a weapon in the battle against illnesses, including arthritis, inflammation, prostate cancer, etc. Although pumpkin seeds were considered as a waste of time and resources, their nutritional value might now play a significant role in the food supply. They are safe to eat on a daily basis and do not have any negative impact on human health [30].

A detailed view of the nutritional composition, bioactive profile, and health benefits of pumpkin bioactive compounds is shown in Table 1, Table 2, Table 3, Table 4 and Table 5.

## 3. Health-Promoting Properties of Cucurbita

### 3.1. Hypoglycemic Properties

Hyperglycemia, which may result from the absence of insulin (DM-type 1) or due to a low response to insulin (DM-type 2), is one of the world’s emerging problems. Chronic hyperglycemia leads to severe complications, such as damage to the eyes (retinopathy), brain (neuropathy), and kidneys (nephropathy) [53]. Diabetes is remarkably spreading worldwide, suggesting a considerable rise to about 82 million sufferers by 2030 [54]. People in third world and middle-class countries suffer from DM as more than 80% of diabetes-associated deaths occur in these regions [55]. Several drugs, such as glucagon-like peptide-1 (GLP-1) analogs, α-glucosidase, metformin, etc. are used to cure DM-2. Nevertheless, these drugs have an effect on longevity, resulting in many adverse side effects. Due to the side effects of anti-hyperglycemic medicines, healthcare providers worldwide focus on the use of herbs and dietary ingredients to cure DM-2 [56].

In Mexico and China, herbal extracts are used to treat hyperglycemia as they usually contain pumpkin [57,58]. In recent years, extensive research has been conducted to study the antidiabetic effects of pumpkin flesh, seeds, and peel [59]. Pumpkin fruit powder was reported to contain antidiabetic properties [60]. In light of this study, it is reported that pumpkin powder tends to enhance the level of insulin in the body, leading to a lower level of glucose. Therefore, it also lowers the risk of kidney damage [61].

The breakdown of complex carbohydrates usually occurs in the small intestine by a-glycosidase, which is present in epithelial mucosa. A-glycosidase is responsible for the breakdown of glycosidic bonds present within complex carbs, and thus increases the blood glucose level [62]. It is often highlighted that the use of pumpkin can lower the activity of a-glycosidase. Moreover, in the same research, it has been reported that pumpkin scavenges ROS and can act as an ACE inhibitor [63]. However, further research is required to understand the effect of pumpkin on alpha-amylase.

Diabetic rats are one of the most commonly used tools to study the antidiabetic effect of any therapeutic agent. Alloxan is a toxin that affects the B-cells of pancreas, and thus leads to diabetes in rats [64]. A case-control study on rats demonstrates the anti-hyperglycemic properties of pumpkin. It further strengthens the research that pumpkin has the tendency to enhance insulin production and lowers the glucose level in the blood. The use of pumpkin in the earlier stages of diabetes may reduce postprandial glycemic levels. Protein-bound polysaccharide (PBPP) in pumpkin tends to lower hyperglycemia in rats and is usually dose-dependent [65]. In 2019, an in vivo study was conducted. Both pumpkin polysaccharide (PPe) and pumpkin polysaccharide hydrolysate (PPe-H) showed their hypoglycemic effect to lower fasting blood sugar levels of rats with DM-2. The diabetes-friendly nature of PPe-H has facilitated the decrease in oxidative stress and stimulated the endogenous GLP-1 secretion [66].

**Figure 3:**
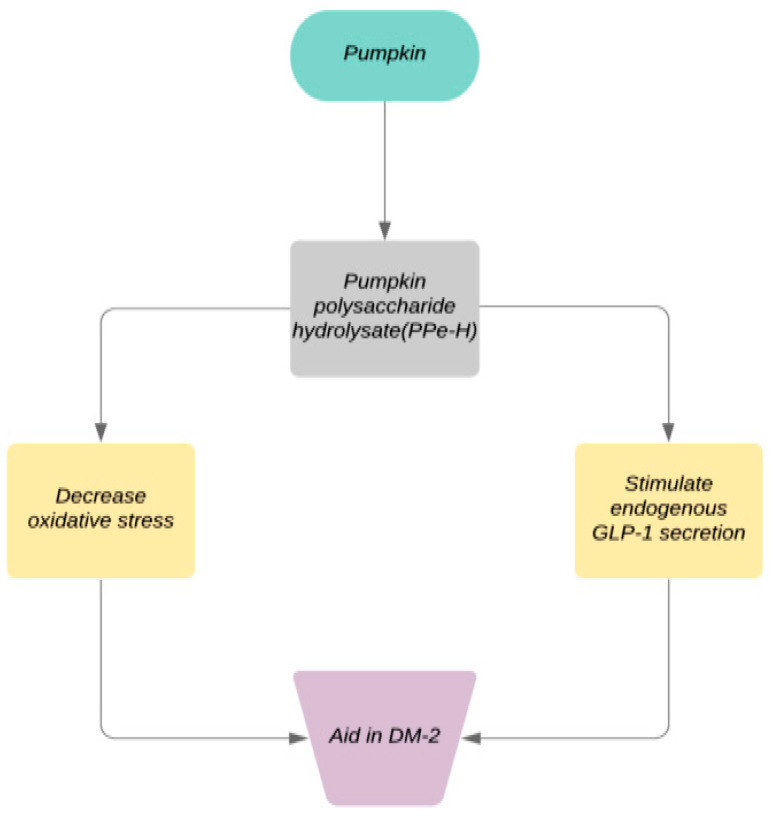
Mechanism of pumpkin polysaccharide hydrolysate in the treatment of DM-2.

Figure 3 illustrates the mechanism of pumpkin polysaccharide hydrolysate in the treatment of DM-2.

Numerous researches established the diabetic-friendly effects of pumpkin [67]. One study reported that pumpkin demonstrates pancreas protective effects as it tends to increase the superoxide dismutase (SOD) levels, and thus protects islet cells against streptozotocin. In another recent study, the combined effect of puerarin and pumpkin polysaccharide was investigated. It was found that both of these compounds tend to alleviate resistance against insulin, followed by PI3K/AKT pathway and the upregulation of Nrf2/HO-1. In addition, they have hypoglycemic properties [66].

**Figure 4:**
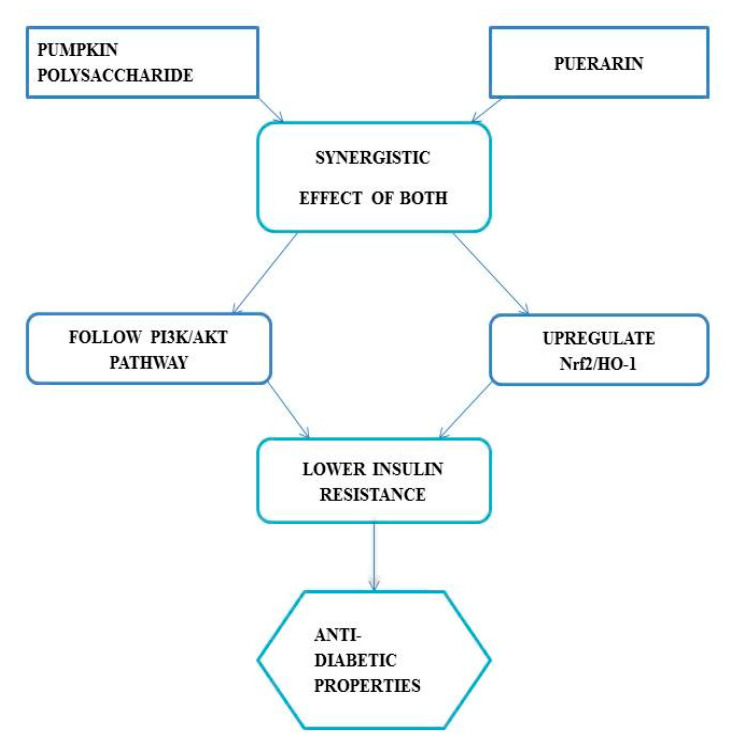
Antidiabetic synergistic effect of both pumpkin polysaccharide and puerarin.

Figure 4 illustrates the synergistic antidiabetic effect of both pumpkin polysaccharide and puerarin.

Hypoglycemic properties of germinated seed oil(s) and seed protein(s) have also been reported. However, ungerminated seeds do not show hypoglycemic properties [68].

In addition to the in vivo studies, several clinical studies have been conducted to understand the efficacy of pumpkin in treating and controlling DM-type 2. It has been reported that fasting blood glucose in people with diabetes is due to the use of juices of pumpkin fruits. Similarly, pumpkin polysaccharide granules have also been proven to control postprandial sugar levels in clinical studies [69,70]. Pumpkin regulates the blood glucose level by promoting the discharge of insulin and preventing many of the complications associated with diabetes [71]. Taking this into account, it is stated that pumpkin has antidiabetic properties and can play a protective role against hyperglycemia in diabetic patients. Nevertheless, researchers could not use these data to successfully report the exact mechanism of action of pumpkin against diabetes.

### 3.2. Anti-Cancerous Properties

A diet rich in oxidants and antioxidants has a significant association with cancer. Diet tends to make the condition worse or better, as cancer is associated with oxidative stress [72]. In 2017, 9.6 million deaths were reported due to all types of cancers. A report by GLOBOCAN estimated that the number of individuals suffering from cancer in 2018 was 18.1 million, and the death rate will be 9.6 million in 2019 [73]. In less developed countries, the rate of stomach, cervical, and liver cancer is high, while in developed countries, breast, lung, and prostate cancer is more common [74,75].

Prevention of cancer is possible using a proper diet and dietary ingredients. Additionally, only 5 to 10% of all cancers are inherited, while the others are due to lifestyle [76]. Pumpkin has been investigated in various researches for its anti-cancerous properties. It has been reported that the risk of several types of cancer, such as breast, rectal, and lung cancer is inversely proportional to pumpkin seeds intake [77]. Among all cancers, deaths from prostate cancer have been reported to be the highest in America [78]. In 2009, a randomized and double-blind study was conducted to investigate the link between prostate cancer and the seed oil of saw palmetto and pumpkin. In this study, 47 patients with the age of 53.3 years were divided into groups. After 3 months, it was observed that pumpkin seed oil combined with saw palmetto oil reduced the serum prostate-specific antigen [79]. For 20 days, an oral intake of 20–40 mg/kg of pumpkin seed oil is considered as effectively useful in the hyperplastic prostate gland by inhibiting testosterone-induced hyperplasia of the prostate [17]. Terpenoids and triterpenoids are reported to have anti-tumor cytotoxic properties [80]. A class of tetracyclic triterpenes, which is cucurbitacin, is further divided into cucurbitacin B (CuB) and cucurbitacin 163 E (CuE) [81]. CuE is reported to have anticancer as well as anti-tumor properties. CuE inhibits the prostate cancer cells by disrupting the structure of actin and vimentin [82]. Cucurbitacin shows anticancer properties by inducing apoptosis via JAK/STAT, PARP, MAPK pathways [83].

In vivo and in vitro studies have demonstrated the inhibitory effects of pumpkin against prostate cancer. Moreover, a study on Sprague Dawley rats reported the inhibition of testosterone-induced hyperplasia using pumpkin seed oil, suggesting the beneficial use of pumpkinseed oil against benign prostatic hyperplasia [84]. Furthermore, a clinical trial demonstrated that the use of pumpkin seed oil in the early phases of prostate cancer is beneficial [79,85].

Pumpkin seeds are a major source of phytoestrogen, such as lignans and isoflavones. Phytoestrogen tends to bind with estrogen receptors (ER) in the female body [86]. A study was conducted to investigate the association between breast cancer and pumpkin seed extract containing phytoestrogens. It was reported that the production of estradiol in MCF7, BeWo, and Jeg3 cells increased, and the reduction in ER-α was observed in MCF7-cells [87]. Essentially, 2S albumins which are proteins present within the seeds of pumpkin, have been reported in a study to have anti-cancerous properties, particularly in the case of breast cancer. MCF7-cells were treated with protein at two different concentrations (10 and 30 µM). The results obtained by DNA showed fragmentation assays and acridine orange staining, in which 2S albumins from pumpkin seeds have the tendency to cause apoptosis in MCF7-cell lines [88].

### 3.3. Neuroprotective Properties

Malnutrition is a common issue worldwide, affecting children with limited calorie and protein(s) intake. Malnutrition usually results in behavioral disorders [89]. It is reported that protein-energy malnutrition (PEM) gives rise to free radical production, usually through lipid peroxidation [90]. Lipid peroxidation is a risk factor associated with brain damage. Free radicals production, such as reactive oxygen species (ROS) damages the brain cells, resulting in severe harmful consequences of PEM [91,92]. In a recent study, the leaves of pumpkin (fluted pumpkin) were used to investigate the brain-protective effect of this herb in PEM-induced rats due to the high antioxidant composition [93]. The seed protein was clearly observed and fluted pumpkin leaves joined together to prevent oxidative damage to brain cells due to PEM.

Organic compounds known as aflatoxins have substantial toxic effects, such as carcinogenic, mutagenic, and hepatotoxic. In addition, they contribute to lipid peroxidation, thus affecting the brain [94]. In 2013, research was conducted to investigate the effect of pumpkin seed oil on aflatoxins-induced toxicity in the brain and other organs. It was reported that pumpkin seed oil has the tendency to treat aflatoxins-induced harmful effects in brain tissues [95].

Depression is the most common brain disorder and is specified as a state in which one loses interest, pleasure, sleep, and eating patterns are disturbed. Similarly, in mood swings, the sufferers may feel guilty and ashamed. In addition, they have poor interest/concentration in daily tasks. According to the 2015 World Health Organization statistics, people suffering from depression were more than 300 million [96]. In a study on fluted pumpkin leaves, it was reported that they can be a useful tool in treating depression and convulsions due to their muscle relaxant properties, particularly in hydroethanolic leaf extract [97]. Moreover, another report demonstrated the antidepressant effects of pumpkin in supporting the treatment of depression [98].

Furthermore, a recent study highlighted that several nutritional deficiencies result in the progression of diabetes, such as vitamin B, amino acids, and omega 3-fatty acids [99]. Tryptophan is a fundamental component of pumpkin seeds, associated with depression relief and social anxiety disorder treatment [100,101]. Pumpkin seeds contain high amounts of tryptophan, 576 mg per 100 g, which lead to a form of serotonin (neurotransmitter), thus aiding in depression [102]. A study was conducted to assess the antidepressant properties of pumpkin seeds. The effect of raw and processed pumpkin was evaluated by inducing depression in rats via injecting methyl isobutyl ketone. The impact of natural and processed pumpkins was assessed. It was reported that both of these pumpkin seed extracts have antidepressant activities and are used as a helpful alternative to antidepressants that have adverse side effects [103].

### 3.4. Liver Disease Preventive Properties

In the past, several researchers reported the liver-protective effects of pumpkin. In 2005, a study was conducted on male Sprague Dawley rats, which were administered a low protein diet for 5 days to produce liver dysfunction. Thereafter, they were administered CCl4 injections, which resulted in significantly higher levels of four liver enzymes; aspartate transaminase (AST), alkaline phosphatase (ALP), alanine transaminase (ALT), and lactate dehydrogenase (LD). One group was administered with pumpkin seed protein isolate, which lowered the level of both aforementioned enzymes, suggesting the beneficial role of pumpkin in treating liver dysregulation [104]. In a similar study which was carried out a year later (2006), the same results were reported in addition to antioxidative effects [105]. In another study performed on rats with CCl4, the same hepatoprotective effect of pumpkin seeds protein isolate was mainly reported by enhancing antioxidant activity and decreasing liver enzymes [106]. In a similar study, acetaminophen was used rather than CCl4 to produce liver injury, and the results were the same as reported earlier [107]. Another research, which was conducted to understand the effect of aqueous leaf extract of fluted pumpkin on anemia, also reported the same results. In this case, 50 mg/kg of aqueous leaf extract of fluted pumpkin has the tendency to regulate ALT and AST [108]. All of these researches support the fact that pumpkin seed protein isolates have the tendency to attenuate the high level of liver enzymes (ALT, AST, ALP, LD) when liver injury is due to a low protein diet or malnutrition. In 2015, Farid et al. reported the same alterations in these liver enzymes with the use of pumpkin [109].

Non-alcoholic fatty liver disease (NAFLD) further leads to atherosclerosis, and CVD is a major health issue worldwide that leads to mortality and morbidity [110]. To cure the NAFLD as well as the onset of NAFLD, intake of fats and the type of fat present in one’s diet play an important role [111,112]. As mentioned above, pumpkin seed oil is rich in unsaturated fatty acids that comprise about 80% of enriched phytochemicals [113].

In 2015, a study was conducted on APOE*-3Leiden (E3L) mice, which is the most suitable model to study NAFLD [114]. Cocoa butter and a cholesterol rich diet were administered to E3L mice, and then they were administered virgin (VIR) or refined (REF) pumpkin oil, since VIR is reported to strongly reduce hepatic inflammation and steatosis [114]. The pumpkin seed oil has also shown its beneficial role in the treatment of oxidative stress as well as in the fatty liver among rats administered with high sugar (fructose) diet [115]. Enhancement of fatty liver to steatohepatitis may be interrupted using nanoemulsions of pumpkin seed oil [116]. Nanoemulsions are dispersions that increase the absorption of drugs, resulting in low medicine use [117]. In addition to nanoemulsions, the formulation of niosomes with PSO is also very beneficial, with a shelf life of 90 days at 30 °C [118]. Therefore, the use of pumpkin seed oil nanoemulsions may act as a promising tool in the future to enhance the efficacy of medications.

Aspartame, which is often used in more than 60 countries worldwide as a sweetener, has also been found to be associated with hepatotoxicity, thus affecting liver functioning [119]. A study was conducted to assess the effect of pumpkin seed oil (PSO) on aspartame. It was reported that supplementation of PSO with water for 4 weeks at a dose of 4 mL/kg can relief the negative effects of aspartame and protect the liver. Through this PSO supplementation, globulin, ALP, ALT, AST, bilirubin, and albumin were maintained [120]. Moreover, free polyphenols of pumpkin are known to have the highest hepatoprotective properties as compared to bound polyphenols [121].

### 3.5. CVD Preventive Properties

Although the death rate associated with CVD has been controlled during the past years, in the US, heart disease is 1st, while stroke remains the 5th leading cause of death [122]. Of note, the aforementioned diabetes has been associated with cardiovascular diseases, which is the main cause of death worldwide due to DM [123]. It is a life-threatening issue worldwide, as common and most usual risk factors are present in young generations, particularly in developing countries [124]. The most common risk factor associated with CVD is hyperlipidemia, the number 1 cause of death worldwide [81].

Several researches have demonstrated an inverse relation between the Mediterranean diet and CVD [125,126]. Cucurbitaceae species is a segment of the Mediterranean diet, and a number of studies have been conducted on the species to understand its association with CVD [127]. It has been reported that unsaturated fats lower the risk of several heart diseases, while saturated fats promote this risk (Demaison and Moreau 2002). In light of this data, a study was conducted in which the level of HDL was increased, while the level of LDL lowered in correspondence to a diet high in mono and polyunsaturated fatty acids [128]. Followed by this available data, another research was conducted in 2011, in which pumpkin oil as well as olive oil were used to rate their impact on serum lipoprotein levels due to their high PUFA and MUFA content. In contrast to the control group, both olive oil and pumpkin oil affected the serum lipid profile. However, the effect of olive oil was significantly higher than pumpkin seed oil. As a result, this confirms the role of pumpkin seed oil in treating heart problems, and further research can be carried out on pumpkin seed oil [129].

Pumpkin affects serum lipid levels directly and can also affect them indirectly. A research was conducted on Lohmann Brown Lite hens, and they were administered with a diet based on flaxseed and pumpkin oil. It was reported that eggs laid by the hens with a pumpkin diet had the highest MUFA content, lower PUFA, lower myristic acid, and other saturated fatty acids content [130]. However, further investigation is required as the earlier increase in myristic acid content was reported in eggs after pumpkin supplementation [131].

A similar study was conducted in 2015, where pumpkin cake was prepared with pumpkin extract (5, 10, and 15%) and wheat flour (72%). Pumpkin cake was high in fiber (8%), carotenoid (41 mg/100 g), and ash (6.45%) compared with the control cake, which was made of wheat. On a biological basis of analysis, it was reported that pumpkin meal lowers the cholesterol and LDL level and increases the HDL level in a dose-dependent manner [132]. Other researches also support these findings, in which rats with hypercholesterolemia were supplemented with pumpkin seeds. It was reported that PS resulted in a decrease in LDL, whereas an increase in HDL level lowered the level of cholesterol [133,134]. In addition to affecting the atherogenicity and blood lipid profile, pumpkin also has a beneficial role in kidney functioning [135].

When the body is in hypercholesterolemic situation, dysfunctioning of the endothelial layer occurs, leading to the enhanced level of vascular cell adhesion molecule (VCAM). This occurs due to the activation of reactive oxygen species (ROS), which reduces nitric oxide formation and stops LDL oxidation. Due to the lower levels of nitric oxide (NO), oxidization of LDL will occur, which is responsible for all this VCAM enhancement mechanism [136]. Pumpkin seed powder contains 2.6% arginine, which is the precursor of NO, associated with BP maintenance, apoptosis, myocardial function, and inflammatory response [137]. An in vivo study designed to investigate the effect of pumpkin seed extract (mainly arginine) reported that pumpkin seed extract supplementation in rats with hyperlipidemia could enhance the expression of NO production due to the presence of arginine. Moreover, due to the production of NO, oxidation of LDL is attenuated, leading to the decreased expression of VCAM [138]. Therefore, it can be stated that modifying the lifestyle and adapting the regular use of pumpkins can be a useful dietary strategy to treat hypercholesterolemia.

### 3.6. Other Health-Related Properties

In addition to all of the aforementioned properties, pumpkin has been reported to play an important role in inflammatory diseases, such as arthritis due to its anti-inflammatory properties [139,140,141].

The worldwide male population above 50 years mostly suffers through an outgrowth of stromal and epithelial cells, usually in the periurethral area and in the transition zone [142,143]. Many researches support the claim that pumpkin seed extract has also been reported to overcome lower urinary tract symptoms (LUTS) associated with benign prostatic hyperplasia (BPH), such as saw palmetto and prazosin [144,145]. In addition to treating LUTS, pumpkin administered for a whole month at a 14 mg/kg dose is a really useful strategy in controlling testosterone-induced hyperplasia [17]. Nevertheless, the data are not sufficient, and further researches are required as many consequences of pumpkin intake, in this case, are neglected in these researches.

Currently, although antimicrobial drugs are available, the focus of scientists is to develop dietary strategies to treat these infections as the use of these antimicrobes can lead to drug resistance. At a concentration of 2% (*v*/*v*), pumpkin seed oil has the tendency to treat Staphylococcus aureus, Salmonella enterica, Escherichia coli, Klebsiella pneumonia, Acinetobacter baumannii, Candida albicans, and Serratia marcescens [60]. Three hundred and seventy five milligrams of pumpkin seed oil can be useful to treat Mycosphaerella arachidis, Fusarium oxysporum, and Botrytis cinerea [146]. Similarly, proteins from pumpkin (>2 mM) in their purified form can control fungal growth [147]. Likewise, yeast growth has been known to be inhibited by three types of pumpkin proteins named MAP11, MAP4, and MAP2. However, MAP2 and MAP4 are not very useful in treating Gram-negative bacteria [148]. Therefore, pumpkin is a food of great antimicrobial properties and its use should be encouraged by people living in areas where they are more susceptible to any kind of microbial infection.

Pumpkin seeds have also shown anti-ulcerative properties by protecting gastric mucosa in a dose-dependent manner [149]. Wound healing through more effective and cost effective strategies is a constant focus of researchers, as medicines seem to be insufficient for the healing process [150,151]. High amounts of plant sterols, tocopherols, and PUFA present in pumpkin seed oil positively affect wound healing [14]. Similar findings that are related to pumpkin peel are also reported [152].

During the past years, male reproduction has been affected due to changes in lifestyle and some tragedies [153]. As pumpkins tend to attenuate lipid peroxidation and lower oxidative stress, it is a valuable tool to improve testicular properties and enhance reproduction [154,155]. A recent study conducted on fluted pumpkin seed (FPS) extract reported that 40 days of supplementation of 40 mg/kg bodyweight of FPS extract could enhance the number of spermatids, spermatocytes, and spermatogonia as well as protect against oxidative cell damage [156]. Therefore, FPS extract can be used as spermatocytes activator after chemotherapy. However, the effect is still uncertain, thus further investigation is required to understand the effect of pumpkin on serum parameters and testes [157].

## 4. Conclusions and Recommendations

Pumpkin belongs to the family Cucurbits, an economical vegetable that is globally cultivated. Different pumpkin fruit parts (seeds, peels, and flesh) are rich sources of micro and macro nutrients, including carbohydrates, fiber, amino acids, MUFA, PUFA, tocopherol, and carotenoids. The presence of various bioactive phytochemical compounds in pumpkin showed health-promising properties and may be used as an ingredient of choice in functional foods and pharmaceutical products. All of these components have an essential role in the body’s normal mechanism, and thus can aid in treating several diseases as functional therapeutic agents. Previous studies confirmed the significant role of pumpkins in managing and treating diabetes, cancer, liver disorders, cardiovascular diseases, and depression. Additionally, the use of cucurbits species as antioxidant, antimicrobial, anti-inflammatory, and anti-ulcerative properties has also been reported by researchers. Moreover, these bioactivities can be improved using environmental stimulators, such as atmospheric CO_2_ and laser

Although previous studies demonstrate the therapeutic uses of pumpkins in major chronic diseases, further research is still required. The effect of pumpkin on a-amylase and the pathway of this climber to exhibit hypoglycemic properties have not yet been reported. Data on the role of pumpkin in prostate and breast cancer are not sufficient, and minimal investigations are conducted on rectal, stomach, and lung cancer. Moreover, pumpkin in spermatogenesis can be used as an effective strategy after chemotherapy, where males suffer from reproduction disorders. In addition to all of these therapeutic effects, pumpkin meals can be used in poultry to produce healthy eggs due to the high MUFA and low saturated fatty acid content of pumpkin. Furthermore, an appropriate collaboration of researchers and industrialists is required to produce pumpkin-based useful products. Finally, awareness among the masses should also be emphasized in regard to the nutritional and therapeutic uses of pumpkin as an ingredient in the daily diet.

## Figures and Tables

**Table 1:** Basic nutritional composition of pumpkin peel, fruit, and seed.

Nutrient	Pumpkin Peel	Pumpkin Fruit	Pumpkin Seed
	(Value/100 g)	(Value/100 g)	(Value/32.25 g)
Energy	520.78 kJ	109 kJ	NR
Water	89.527 mg	91.6 g	1.69 g
Lipids	1.650 mg	0.1 g	15.82 g
Protein	14.670 mg	1.0 g	9.75 g
Ash	7.317 mg	0.8 g	1.54 g
Dietary Fiber	13.383 mg	0.5 g	1.94 g
Carbohydrates	12.407 mg	6.5 g	3.45 g
Total Sugars	7.633 mg	2.76 g	NR
Calories	NR	26 kcal	180.28 kcal
Carotene, beta	NR	3100 µg	NR
Carotene, alpha	NR	4016 µg	NR
Reference	Amin et al. [20]	USDA [31]	Mateljan [32]

NR: Not reported.

**Table 2:** Mineral composition of pumpkin peel, fruit, and seed.

Nutrient	Pumpkin Peel	Pumpkin Fruit	Pumpkin Seed
	(mg/100 g)	(mg/100 g)	(mg/32.25 g)
Calcium	1.360	21	14.84
Iron	4.004	0.8	2.84
Magnesium	3.353	12	190.92
Phosphorous	1.419	44	397.64
Potassium	687.467	340	260.90
Sodium	9.652	1.0	2.26
Zinc	0.150	0.32	2.52
Copper	0.025	0.127	0.43
Manganese	0.360	0.125	1.47
Selenium	NR	0.3 µg	NR
Reference	Amin et al. [20]	USDA [31]	Mateljan [32]

NR: Not reported.

**Table 3:** Vitamin composition of pumpkin fruit and seed.

Nutrient	Pumpkin Fruit	Pumpkin Seed	
	(mg/100 g)	(mg/100 g)	(mg/32.25 g)
Vitamin A	0.426	0.019	0.0015
Vitamin C	9.0	0.3	0.61
Vitamin B1	0.05	0.034	0.09
Vitamin B2	0.11	0.052	0.05
Vitamin B3	0.6	0.286	1.61
Vitamin B5	0.298	0.056	0.24
Vitamin B6	0.061	0.037	0.05
Vitamin B9	0.016	0.009	0.0187
Vitamin E	1.06	NR	0.70
Vitamin K	0.001	NR	0.0023
Reference	Amin et al. [20]	USDA [31]	Mateljan [32]

NR: Not reported.

**Table 4:** Presence and absence of various bioactive compounds in different cultivars of pumpkin [33].

		Cultivar											
		Bambino	Hokkaido	Gomez	Melonowa Żółta	Porcelain Doll	Blue	Kuri	Buttercup	Jumbo Pink Banana	Jarrahdale	Marina di Chioggia	Green Hubbard
Carotenoids	Zeaxanthin	+	+	+	+	+	+	+	+	+	+	+	+
	Lutein	+	+	+	+	+	+	+	+	+	+	+	+
	β-carotene	+	+	+	+	+	+	+	+	+	+	+	+
Phenolic acids	Gallic acid	+	+	+	+	+	+	+	+	+	+	+	+
	Protocatechuic acid	+	+	+	+	+	+	+	+	+	+	+	+
	4-Hydroxy-benzoic acid	+	+	+	+	+	+	+	+	+	+	+	+
	Vanillic acid	+	+	+	+	+	+	+	+	+	+	+	+
	Chlorogenic acid	+	+	+	+	+	+	+	+	+	+	+	+
	Caffeic acid	+	+	+	+	+	+	+	+	+	+	+	+
	p-coumaric acid	−	+	−	+	+	+	+	+	+	+	+	+
	Ferulic acid	+	+	+	+	+	+	+	+	+	+	+	+
	Sinapic acid	+	+	−	+	−	+	+	−	+	−	+	+
Flavonols	Rutin	+	+	+	+	+	+	+	+	+	+	+	+
	Kaempferol	−	+	+	+	−	+	+	−	+	−	+	+
	Isoquercetin	+	+	+	+	+	+	+	−	+	+	+	−
	Astragalin	+	−	−	+	+	+	+	−	−	+	+	−
	Myricetin	+	+	−	−	+	+	+	+	+	−	−	+
	Quercetin	+	+	+	+	+	+	+	+	−	+	+	+
Tocopherols	α-tocopherol	+	+	+	+	+	+	+	+	+	+	+	+
	γ-tocopherol	+	+	+	+	+	+	+	+	+	+	+	+

+: Present; −: Not detected.

**Table 5:** Molecular formula, molecular weight, structures, and health effects of various bioactive compounds of pumpkin.

Compound	Molecular Formula	Molecular Weight	Structure	Health Effects	Reference
Zeaxanthin	C_40_H_56_O_2_	568.9	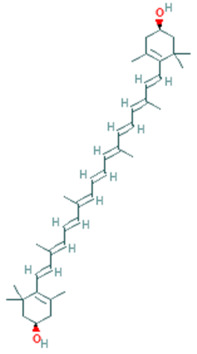	Promotes Eye Health	[34]
Lutein	C_40_H_56_O_2_	568.9 g	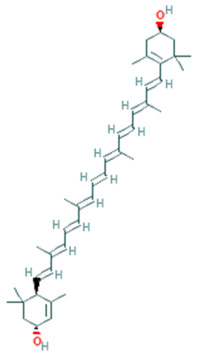	Promotes Eye Health	[34]
β-carotene	C_40_H_56_	536.9	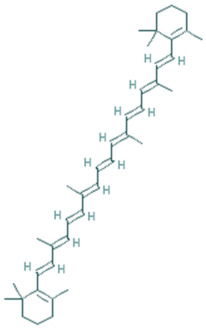	An important vitamin source for humans	[35]
Gallic acid	C_7_H_6_O_5_	170.12	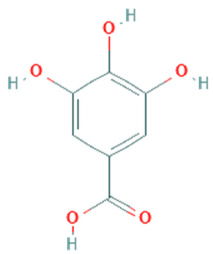	Performs excellent activities for gastrointestinal, neuropsychological, metabolic, and cardiovascular disorders	[36]
Protocatechuic acid	C_7_H_6_O_4_	154.12	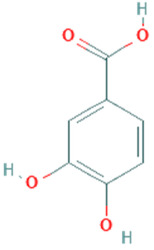	Strong antioxidant, best known for cardiovascular-protective effects	[37]
4-Hydroxy-benzoic acid	C_7_H_6_O_3_	138.12	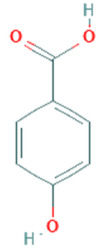	Antisickling, analgesic, and anti-inflammatory properties	[38]
Vanillic acid	C_8_H_8_O_4_	168.15	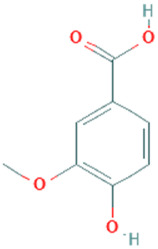	Antioxidant; potentially reduces oxidative stress	[39]
Chlorogenic acid	C_16_H_18_O_9_	354.31	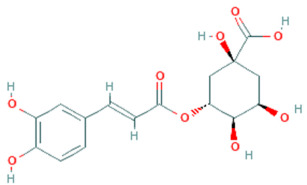	Used as a treatment for metabolic syndrome, including antioxidant, anti-inflammatory, antilipidemic, antidiabetic, and antihypertensive activities	[40]
Caffeic acid	C_9_H_8_O_4_	180.16	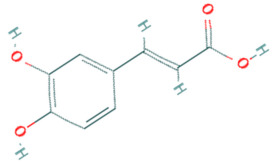	Possesses strong anti-inflammatory to anticancer effects	[41]
p-coumaric acid	C_9_H_8_O_3_	164.16	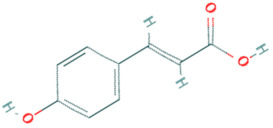	Strong protective effects against acetaminophen-induced hepatotoxicity	[42]
Ferulic acid	C_10_H_10_O_4_	194.18	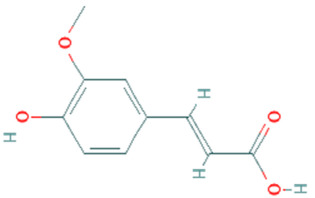	Strong antioxidant, prooxidant, and has strong antibacterial activities	[43]
Sinapic acid	C_11_H_12_O_5_	224.21	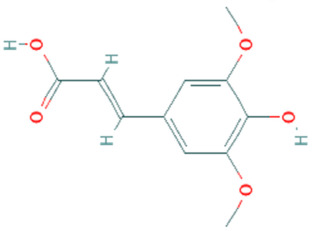	Neuroprotective and might be beneficial in the treatment of Alzheimer’s disease	[44]
Rutin	C_27_H_30_O_16_	610.5	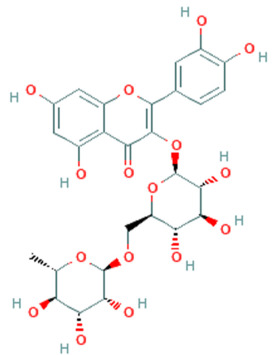	Neuroprotective agent	[45]
Kaempferol	C_15_H_10_O_6_	286.24	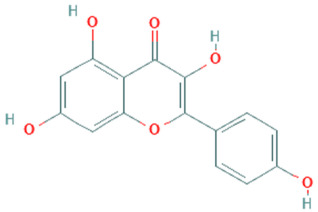	Strong anticancer potential	[46]
Isoquercetin	C_21_H_20_O_12_	464.4	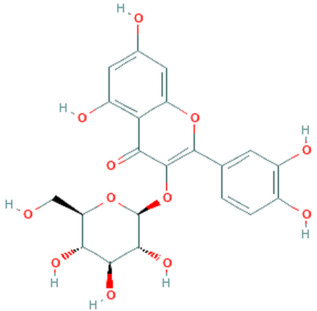	Suppresses colon cancer cell growth	[47]
Astragalin	C_21_H_20_O_11_	448.4	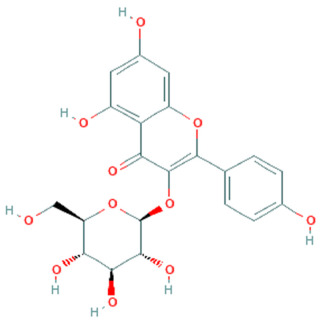	Reduces lipopolysaccharide-induced acute lung injury	[48]
Myricetin	C_15_H_10_O_8_	318.23	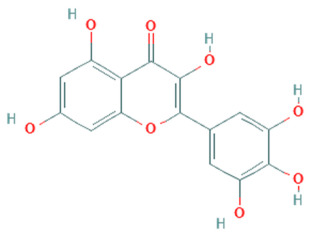	Protects cardiomyocytes from LPS-induced injury	[49]
Quercetin	C_15_H_10_O_7_	302.23	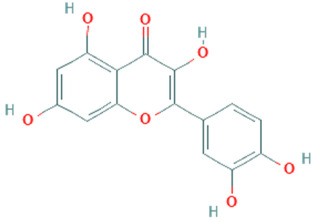	Strong antidiabetic potential	[50]
α-tocopherol	C_29_H_50_O_2_	430.7	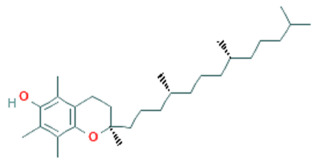	Potential antioxidant	[51]
γ-tocopherol	C_28_H_48_O_2_	416.7	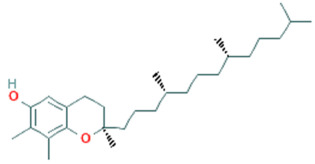	*Y*-tocopherol concentrations are inversely associated with antioxidant exposures	[52]

## Data Availability

Not Applicable.
